# Involvements of p38 MAPK and oxidative stress in the ozone-induced enhancement of AHR and pulmonary inflammation in an allergic asthma model

**DOI:** 10.1186/s12931-017-0697-4

**Published:** 2017-12-29

**Authors:** Aihua Bao, Hong Yang, Jie Ji, Yuqin Chen, Wuping Bao, Feng Li, Min Zhang, Xin Zhou, Qiang Li, Suqin Ben

**Affiliations:** 1 Department of Respiratory Medicine, Shanghai General Hospital, School of Medicine, Shanghai Jiao Tong University, 100 Haining Road, Shanghai, 200080 People’s Republic of China; 2 Unit for Lung and Airway Research, Institute of Environmental Medicine, Karolinska Institutet, PO Box 210, -17177 Stockholm, SE Sweden

**Keywords:** Asthma exacerbation, Ozone, p38 MAPK, Oxidative stress, α-tocopherol

## Abstract

**Background:**

Exposure to ambient ozone (O_3_) increases the susceptivity to allergens and triggers exacerbations in patients with asthma. However, the detailed mechanisms of action for O_3_ to trigger asthma exacerbations are still unclear.

**Methods:**

An ovalbumin (OVA)-established asthmatic mouse model was selected to expose to filtered air (OVA-model) or 1.0 ppm O_3_ (OVA-O_3_ model) during the process of OVA challenge. Next, the possible involvements of p38 MAPK and oxidative stress in the ozone actions on the asthma exacerbations were investigated on the mice of OVA-O_3_ model by treating them with SB239063 (a p38 MAPK inhibitor), and/or the α-tocopherol (antioxidant). Biological measurements were conducted including airway hyperresponsiveness (AHR), airway resistance (Raw), lung compliance (CL), inflammation in the airway lumen and lung parenchyma, the phosphorylation of p38 MAPK and heat shock protein (HSP) 27 in the tracheal tissues, and the malondialdehyde (MDA) content and the glutathione peroxidase (GSH-Px) activity in lung tissues.

**Results:**

In OVA-allergic mice, O3 exposure deteriorated airway hyperresponsiveness (AHR), airway resistance (Raw), lung compliance (CL) and pulmonary inflammation, accompanied by the increased oxidative stress in lung tissues and promoted p38 MAPK and HSP27 phosphorylation in tracheal tissues. Administration of SB239063 (a p38 MAPK inhibitor) on OVA-O3 model exclusively mitigated the Raw, the CL, and the BAL IL-13 content, while α-tocopherol (antioxidant) differentially reduced the BAL number of eosinophils and macrophages, the content of BAL hyaluronan, the peribronchial inflammation, as well as the mRNA expression of TNF-α and IL-5 in the lung tissues of OVA-O3 model. Administration of these two chemical inhibitors similarly inhibited the AHR, the BAL IFN-γ and IL-6 production, the perivascular lung inflammation and the lung IL-17 mRNA expression of OVA-O3 model. Interestingly, the combined treatment of both compounds together synergistically inhibited neutrophil counts in the BALF and CXCL-1 gene expression in the lung.

**Conclusions:**

O_3_ exposure during the OVA challenge process promoted exacerbation in asthma. Both p38 MAPK and oxidative stress were found to play a critical role in this process and simultaneous inhibition of these two pathways significantly reduced the O_3_-elicited detrimental effects on the asthma exacerbation.

## Background

Bronchial asthma is a highly prevalent chronic airway disease affecting nearly 300 million people worldwide [1] and characterized by airway inflammation and airway hyperresponsiveness (AHR). Acute exacerbation of asthma is characterized by severe airflow obstruction, due to the enhanced airway inflammation, hypercontractility of airway smooth muscle and airway wall edema*.* Asthma exacerbations are often triggered by environmental allergens, virus infections and air pollutions [2]. As one of the air pollutants, ozone (O_3_) is a ubiquitous photochemical oxidant and has potential adverse impacts on human health, especially the respiratory system [3]. Exposure to O_3_ not only increases the burden of oxidative stress in lungs [4, 5], but also exerts detrimental effects on respiratory mechanics [6, 7].

In asthmatic patients, O_3_ exposure was found to partially contributes to their exacerbations. Several studies have reported that the elevation of local atmosphere O_3_ level is associated with the average visits of asthmatic patients to emergency departments, implying a causative role for O_3_ in triggering the exacerbation of asthma [8, 9]. Particularly, continuous exposure to O_3_ is very harmful to the asthma patients. However, it is by far not clear how O_3_ influences asthma patients. Understanding the action of O_3_ on asthma exacerbation may offer asthmatic patients with more inclusive advices and potential therapeutic options.

Though numerous animal studies have explored the influences of O_3_ on the airways with acute allergic inflammation, most of them applied O_3_ exposure before or after the challenge process. However, studies have shown that O_3_ interfered with the immune responses during the challenge process of allergy establishment. For examples, Depuydt et al. have proved that O_3_ does not affect the sensitization process but does affect the challenge process [10]. In fact, in a scenario that the exacerbations of asthmatic patients are triggered by ambient O_3_, the challenge process will be subject to the O_3_’s influence. Therefore, under such scenario both the immune response process and the subsequent allergic airway inflammation of these patients are vulnerable to the ozonic effects. For the best of our knowledge, so far there is no comprehensive animal studies to investigate the effects of O_3_ on the pathophysiological features of an allergic asthma model during the challenge process.

To date, the underlying mechanisms for in vivo ozonic effects on exacerbation of asthma remain elusive. Studies have shown that p38 mitogen-activated protein kinas (MAPK) might be involved in this process. For example, Williams et al. reported that p38 MAPK contributes to the O_3_-induced airway hyperresponsiveness (AHR) [11], while Li et al. later demonstrated that p38 MAPK activation in the airway smooth muscle further activated heat shock protein (HSP) 27 and subsequently contributed to the O_3_-increased contractility [12]. On the other hand, other researchers speculated that oxidative stress could be the major player in the action of O_3_, based on the fact that O_3_ exposure elevates the oxidative stress level in lung tissues and airway lumen in both humans [13] and rodents [14]. It is by far not known whether the activation of p38 MAPK and the oxidative stress are involved in the ozonic effects during the challenge process, triggering the asthma exacerbations.

In current study, we exposed an OVA-sensitized asthmatic mouse model to O_3_ during the OVA challenge process to mimic O_3_-induced asthma exacerbation. To further illustrate the underlying mechanisms of ozonic effects on this model, we investigated the biological function of p38 MAPK and oxidative stress using their corresponding inhibitors. This study revealed specific ozonic effects on an allergic asthma model involved p38 MPAK and oxidative stress. Additionally, it led to a possible strategy to attenuate the O_3_-elicited detrimental effects on asthma exacerbation and in other oxidative stress-related inflammatory airway diseases, like chronic obstructive pulmonary diseases.

## Methods

### Animal model

Six-week-old female Balb/c mice weighting 18~20 g were purchased from SLAC Laboratory Animal Co. Ltd. (Shanghai, China) and bred under specific-pathogen-free conditions. The animals were kept on an ovalbumin (OVA)-free diet. The protocol was approved by the Shanghai General Hospital Institutional Review Board (Permit Number: 2010KY047). All surgery was performed under sodium pentobarbital anesthesia, and all efforts were made to minimize suffering.

#### OVA asthmatic mouse model

Mice were sensitized intraperitoneally with 20 μg OVA (Grade V, Sigma Aldrich) on day 1, 14, and challenged via aerosol nebulization with 5% OVA (wt/vol) for 30 min each day from day 24 to day 26 (the control mice received PBS in both steps), as previously described [15].

#### OVA-O_3_ model

During the OVA challenge process, equal number of mice were exposed to 1.0 ppm O_3_ or filtered air for 3 h (h) daily on day 23, 25 (OVA challenge was performed 30 min before O_3_ exposure) and 27, as previously described [15]. This OVA-O_3_ model was established to mimic the scenario in which asthmatic patients are triggered by continuous exposure to relatively high level of O_3_ in the atmosphere. This dose of O_3_ exposure has been adapted by a previous study in which the researchers documented that such level of O_3_ inhalation in mice induced the airway inflammation, but did not change the body weight [16]. To illustrate the specific effects of O3 on the allergic mice, a group of O3-exposed normal mice was set as controls.

#### Chemical inhibitor administration

**Fig. 1:**
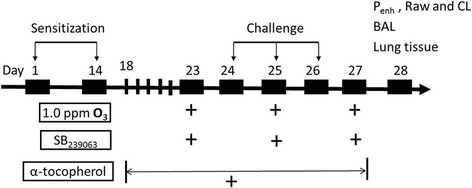
Schematic diagram of the experimental protocol. Mice were sensitized at day 1 and 14, and challenged with OVA or saline at day 24, 25 and 26. Equal number of mice were exposed to 1.0 ppm O_3_ or filtered air for 3 h on day 23, 25 and 27. In a separate experiment, same number of O_3_-exposed asthmatic mice were injected through tail vein with SB239063 (4 mg/kg, diluted with DMSO) prior to each O_3_ exposure, or orally fed by gavage with α-tocopherol (15 IU/kg, diluted in 50% ethanol) for 10 consecutive days (from day 18 to day 27), or received them both. On day 28, measurements were performed including enhanced pause (P_enh_), airway resistance (Raw), lung compliance (CL), cell counts and cytokines in the BALF, mRNA expression of cytokines in the lung tissues, histological evaluation, p38 MAPK-HSP27 signaling by immunoblotting, and oxidative stress

In an independent experiment, mice of the OVA-O_3_ model received further administration of the p38 inhibitor SB239063 and/or the radical scavenger antioxidant α-tocopherol. SB239063 (4 mg/kg) (Sigma Aldrich, St. Louis, MO) dissolved in 3% dimethylsulfoxide (DMSO, resolved with PBS) (0.1 ml) was injected through tail vein 1 h before and 4 h after each O_3_ exposure (control mice received DMSO alone). The dose of SB239063 was adopted based on a previous study where a dose/response experiment was conducted [15]. Alpha-tocopherol (15 IU/kg) (Sigma-Aldrich) dissolved in 50% ethanol (Sigma-Aldrich) in a total volume of 10 μl was delivered via oral gavage twice daily for 10 consecutive days (day 18 to 27, 1 h before O3 exposure or 30 min before OVA challenge from day 23 to 27, and control mice received 50% ethanol alone), as previously described [17]. The detailed experimental protocol was outlined in Fig. 1.

### Airway hyperresponsiveness (AHR)

On day 28, all mice were placed in a whole-body plethysmography (Buxco™, NY) to measure enhanced pause (P_enh_), as described previously [18]. The value was expressed as percentage change from baseline. The log concentrations of methacholine required to increase P_enh_ by 100% from baseline (LogPC_100_P_enh_) was calculated. The decrease in this value in comparison to the normal control indicates the AHR.

### Airway resistance (raw) and lung compliance (CL)

To verify the alterations reflected by Penh, we performed a separate experiment, where the Raw and CL were measured invasively in intact, intubated, anesthetized, spontaneously breathing mice, according to a slightly modified method originally published for rats [19, 20]. Briefly, a pentobarbital (80 mg/kg) anaesthetized mouse was placed and kept warmed in a supine position. After exposure, the tracheal was incised and incubated with a short polyethylene cannula which was fixed by a ligature around the trachea and connected to a heater-controlled pneumotachograph (Hans Rudolph, USA), which linked to a differential pressure transducer (AutoTran, USA) to measure the tidal flow. Another tube filled with water was inserted into the lower third part of the oesophagus and also linked to a pressure transducer (Jialong, Shanghai) to measure the intraesophageal pressure, which was taken as transpulmonary pressure. Respiratory volume was obtained by electric integration of the flow signal. Within a complete respiratory circle, lung resistance (RL), dynamic compliance (Cdyn) and respiratory rate (RR) were measured by integrating the data of airway flow, respiratory volume, and pressure, and recorded every 5 s using MFLab 3.01 software (Shanghai Medical College, Fudan University, China).

### Bronchial alveolar lavage (BAL) collection and cytokine measurements

After overdose sacrifice, bronchoalveolar lavage (BAL) fluid was collected, followed by total and differential leukocytes counts, as previously described [15]. Concentrations of IFN-γ, IL-6, IL-13 and hyaluronan (HA) in BAL fluid were measured with commercial ELISA kits (R&D Systems China Co. Ltd., Shanghai, China), following the manufacturer’s protocols.

### Lung histology

The left lung lobe was fixed in 10% neutral-buffered formalin solution and embedded into paraffin. Lung sections (5 μm) were undergone hematoxylin and eosin (H&E) staining. The infiltration of inflammatory cells in peribronchial area and perivascular area were evaluated according to a 0~3 scoring system as previously described [18, 21].

### Immunoblotting

The phosphorylation of p38 MAPK and HSP27 in tracheal tissues was measured by immunoblotting, as previously described [15]. Briefly, total protein was extracted from tracheal tissues using RIPA buffer (Cell signaling technology, MA). The protein was separated by SDS–PAGE (Bio-Rad Laboratories, Inc., CA) electrophoresis, and transferred to PVDF membranes. The membranes were blocked and incubated with primary antibodies against phosphorylated (phospho-) p38 MAPK and phospho-HSP27, and then stripped and reprobed for total p38 MAPK and total HSP27 (all antibodies from Cell Signaling Technology Inc.™). The binding of the primary antibody was detected by infrared dye-conjugated secondary antibodies and Odyssey® system (Li-Cor, Inc., NE), and bands were quantified with densitometry.

### RT-qPCR

As previously described [15], total RNA was extracted in lung tissue and used to generate cDNA. Transcript levels were determined using SYBR Green PCR Master Mix Reagent (Qiagen, Stockach, Germany). The relative abundance of mRNA of IL-5, IL-17, TNF-α and CXCL-1 was normalized to β-actin. The sequences of primers used in the PCR (synthesized by Invotrigen, Thermo Fisher Scientific Inc., MA) are listed in Table 1.

**Table 1:** Sequences of primers used in RT-qPCR

primer	Forward primer	Reverse primer
IL-5	5’-CCATGCAGAGTCCTCAGAAC AA-3’	5′- TTACTGGAA AGGCCCAAG CA-3’
IL-17	5′- CCTGGCGGCTACAGTGAAG-3’	5′- TTTGGACACGCTGAGCTTTG-3’
TNF-α	5’-AGCCGATGGGTTGTACCTTGTC TA-3’	5’-TGAGATAGCAAATCGGCTGACGGT-3’
CXCL1	5’-TGGCTGGGATTCACCTCAAGAACA-3’	5’-TGTGGCTATGACTTCGGTTTGGGT-3’

### MDA and GSH-Px analysis

Malondialdehyde (MDA) level and glutathione peroxidase (GSH-Px) activity in the lung tissue homogenates were measured using their corresponding substrates (both from Nanjing Jiancheng Bioengineering Institute, Jiangsu, China) through a spectrophotometry-based method, as previously described [15].

### Statistical analysis

Data were analyzed as mean ± SEM. One-way ANOVA and *S-N-K* (*Student-Newman-Keuls*) post hoc test were performed for comparisons in multiple groups. The synergistic effects were detected by univariate analysis of general linear model, using SPSS 17.0 program (IBM Corp., Armonk, NY). A *p* value less than 0.05 was considered statistically significant.

## Results

### Effects of O_3_ exposure on the allergic asthma model

**Fig. 2:**
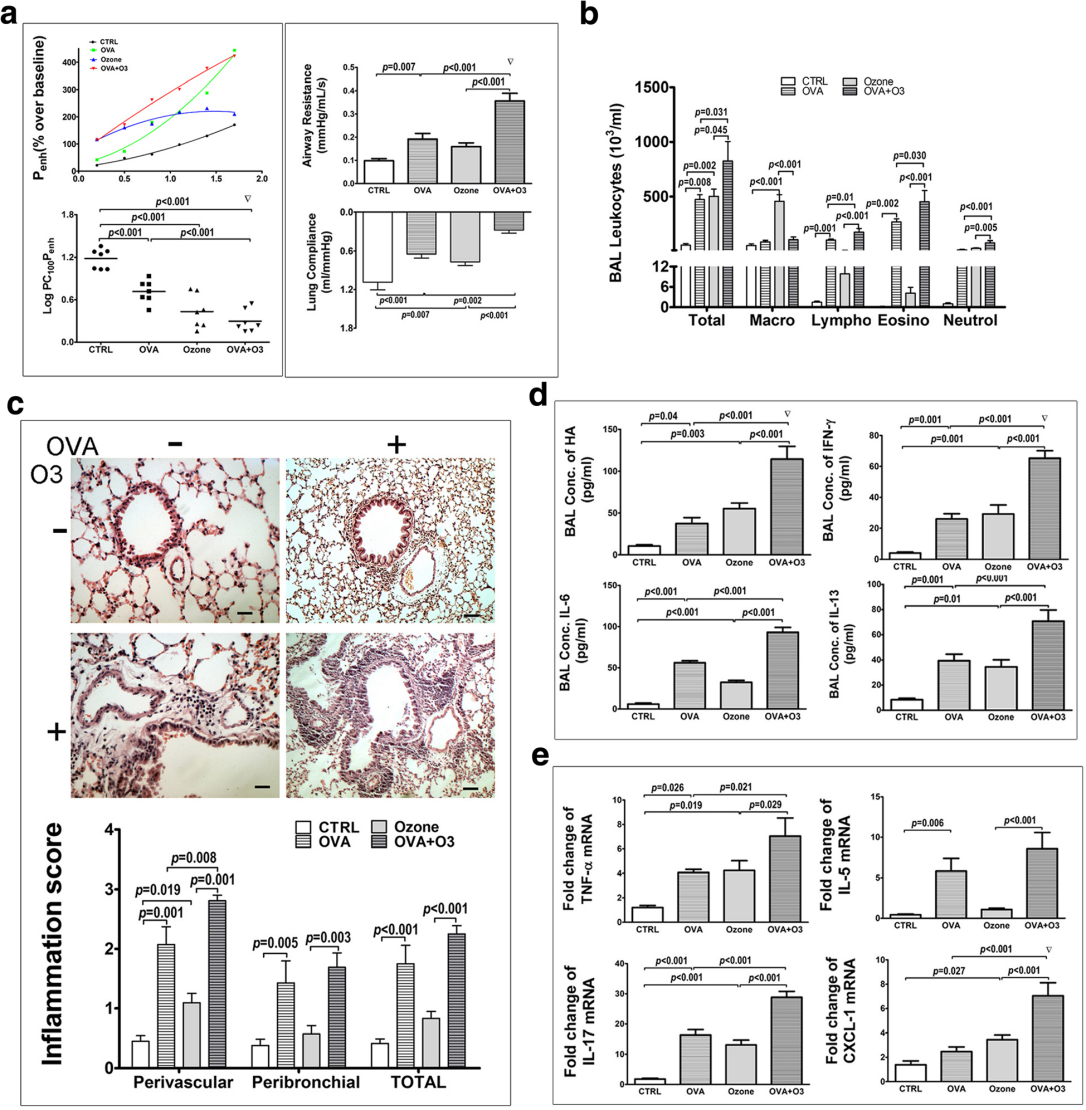
Effects of O_3_ exposure on the allergic asthma model. **a** The lung function was assessed by the AHR in the left panels with the dose-response curves of P_enh_ (top) and the log concentrations of methacholine required to increase P_enh_ by 100% from baseline (LogPC_100_P_enh_) (bottom), the airway resistance (top right) and the lung compliance (bottom right). **b** The total and differentiated cell counts in the BALF. **c** The representative images of H&E stained lung sections (top) and histological inflammation scores (bottom) were shown. **d** cytokines (IL-6, IL-13 and IFN-γ) and HA concentration in the BALF. **e** mRNAs expression of TNF-α, IL-5, IL-17 and CXCL-1 in the lungs. Data are shown as mean ± SEM, *n* = 7 in each group. Synergistic effect of O_3_ and OVA, ▽:*p* < 0.05

First, we evaluated the lung functions/mechanics upon O_3_ exposure and the OVA challenge. Comparing to the normal controls, the OVA-sensitized/challenged mice (OVA model) exhibited AHR (decreased LogPC100Penh), increased airway resistance (Raw), and decreased lung compliance (CL) (Fig. 2a). O_3_ exposure alone had similar effects with enhanced AHR and decreased CL, but the increase in Raw was not significant. O_3_ exposure on the OVA-sensitized/challenged mice (OVA-O_3_ model) further worsen the situations with a synergistic effect on the elevation of both AHR and Raw, while the CL was significantly lower than either OVA challenge or O_3_ exposure alone (Fig. 2 a).

The cell infiltration into the lung was analyzed by the total and differential cell counting in the BALF (Fig. 2 b). Mice of OVA model had higher number of total leukocytes, lymphocytes, eosinophils and neutrophils. O_3_ exposure itself increased the number of total leukocytes, macrophages, neutrophils. In the OVA-O_3_ model, the number of the total leukocytes and all the sub-types were further elevated except macrophages.

Figure 2c showed the histological evaluation of the lung sections and the corresponding inflammation score. It was clearly seen that mice of OVA model had higher inflammation score in the perivascular and peribronchial area. O_3_ exposure increased inflammation score of perivascular but not peribronchial area in the lung sections of both control mice and OVA model.

We further analyzed the selected cytokines (IL-6, IL-13 and INF-γ) and hyaluronan (HA) content in the BALF. As shown in Fig. 2d, all these cytokines and HA were elevated in the OVA model as well as in the O_3_ exposed normal mice; they were further increased in the OVA- O_3_ model. Amongst them, a significant synergistic effect of O_3_ and OVA was observed on the increase in HA and IFN-γ.

Additional cytokines including TNF-α, IL-5, IL-17 and CXCL-1 were measured by RT-PCR assay on their mRNA expressions in the lungs. Figure 2e showed that mice of OVA models exhibited higher mRNA expression of TNF-α, IL-5 and IL-17. Slightly different from OVA model, O_3_ exposure to the control mice or OVA model mice upregulated TNF-α and IL-17 expression, but not IL-5. Interestingly, O_3_ exposure specifically up-regulated the CXCL-1 expression in the lungs of control mice and OVA model mice, where a synergistic effect was observed in the OVA-O_3_ model.

### Define the involvements of p38 MAPK and oxidative stress in OVA-O_3_ mouse model

**Fig. 3:**
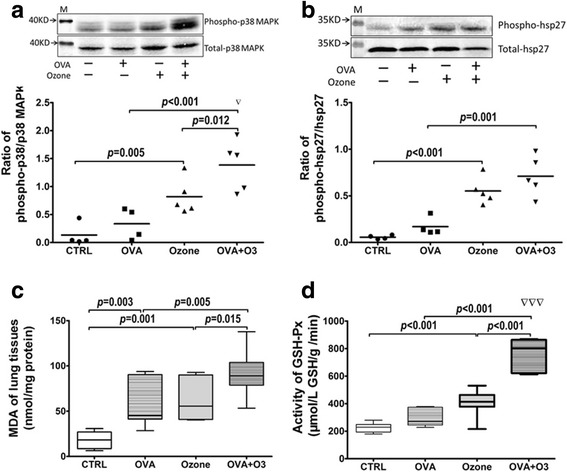
The mechanism(s) of action of O_3_ on OVA-sensitized mice. O_3_ exposure affected the phosphorylation of p38 MAPK (**a**) and its downstream HSP27 (**b**) in tracheal tissues; *n* = 4 in control and OVA groups, *n* = 5 in ozone and OVA + O3 groups. The oxidative stress was evaluated by the content of malondialdehyde (MDA) (**c**) and the activity of glutathione peroxidase (GSH-Px) (**d**) in lung homogenates (*n* = 7 in each group). Data are shown as mean ± SEM. Synergistic effect of O_3_ and OVA, ▽:*p* < 0.05; ▽▽▽: *p* < 0.001

It is known that p38 MAPK-HSP27 cascade and the oxidative stress are highly involved in the O_3_-induced lung inflammation in normal mice [11, 12, 14]. Thus, we anticipated that these two factors may also play roles in O_3_-induced asthmatic exacerbation in our OVA-O_3_ model. Accordingly, we set out to analyze the p38-HSP27 signaling by measuring the phosphorylation of both p38 MAPK and HSP27 in tracheal tissues, and to assess the level of oxidative stress in lung tissues. In comparisons with normal controls, mice in OVA model have similar phosphorylation level of p38 MAPK and HSP27, however, O_3_ exposure increased the phosphorylation level of p38 MAPK and HSP27 in both control mice and the OVA model mice. A synergistic effect of OVA and O_3_ was observed on the phosphorylation of p38 MAPK (Fig. 3a and b).

Oxidative stress was assessed by measuring the MDA level and the GSH-Px activity. Figure 3c showed that the MDA level was increased in mice of OVA model and O_3_ exposed normal mice; it was further elevated in OVA-O_3_ model. The GSH-Px activity, on the other hand, was significantly increased only upon O_3_ exposure, and such increase was found to be synergistic in the O_3_-OVA model (Fig. 3d).

### Effect of SB239063 and α-tocopherol treatment on OVA-O_3_ mouse model

To further determine whether p38 MAPK activation or the presence of oxidative stress or both is indeed contributing to the O_3_-induced lung inflammation in the OVA-O_3_ model, we applied a p38 MAPK inhibitor, SB239063, and/or an oxygen radical-scavenger, α-tocopherol to the mice of OVA-O_3_ model. Their biological influences on the activation of p38 MAPK-HSP27 cascade and the generation of oxidative stress were further examined.

**Fig. 4:**
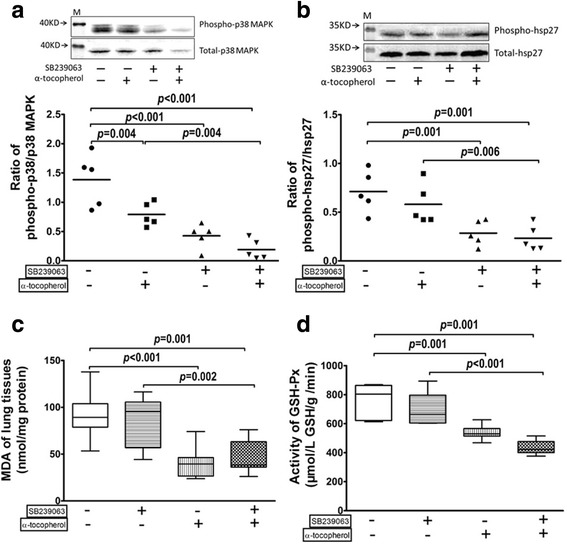
Effect of SB239063 and α-tocopherol treatment on OVA-O_3_ mice model. Equal numbers of OVA-O_3_ mice were received tail vein injection of SB239063 (4 mg/kg) (DMSO as control), or oral feeding of α-tocopherol (15 IU/kg) (50% ethanol as control), or both. The phosphorylation of p38 MAPK (**a**) and HSP27 (**b**) in tracheal tissues were measured by immunoblotting; the MDA content (**c**) and the GSH-Px activity (**d**) in lung homogenates were analyzed to assess the oxidative stress. Data are shown as mean ± SEM, *n* = 5 in each group

As shown in Fig. 4a, administration of SB239063 alone on the OVA-O_3_ model significantly decreased the phosphorylation of p38 MAPK in tracheal tissues, and so as the administration of α-tocopherol alone. The combination treatment further decreased the p38 phosphorylation. Similarly, the downstream HSP27 phosphorylation in tracheal tissues was reduced by SB239063 alone and the co-treatment of SB239063 with α-tocopherol (Fig. 4b); However, the administration of α-tocopherol alone did not affect the HSP27 phosphorylation (Fig. 4b).

In terms of the oxidative stress, administration of α-tocopherol alone as well as its combination with SB239063 significantly decreased the MDA content in the lung tissues, while SB239063 alone had no effect (Fig. 4c). A similar pattern was observed on the change of GSH-Px activity in the lung tissues, where only α-tocopherol was able to reduce the GSH-Px activity with/without the presence of SB239063 (Fig. 4d).

### Role of p38 MAPK and oxidative stress in the ozonic effects on OVA challenged mice

After utilizing chemical inhibitors selectively targeting the p38 MAPK and oxidative stress, we conducted a series of functional, pathological and biochemical measurements to explore whether these two pathways play a role in mediating the O_3_-induced hyper-inflammation on OVA-sensitized/challenged mice.

**Fig. 5:**
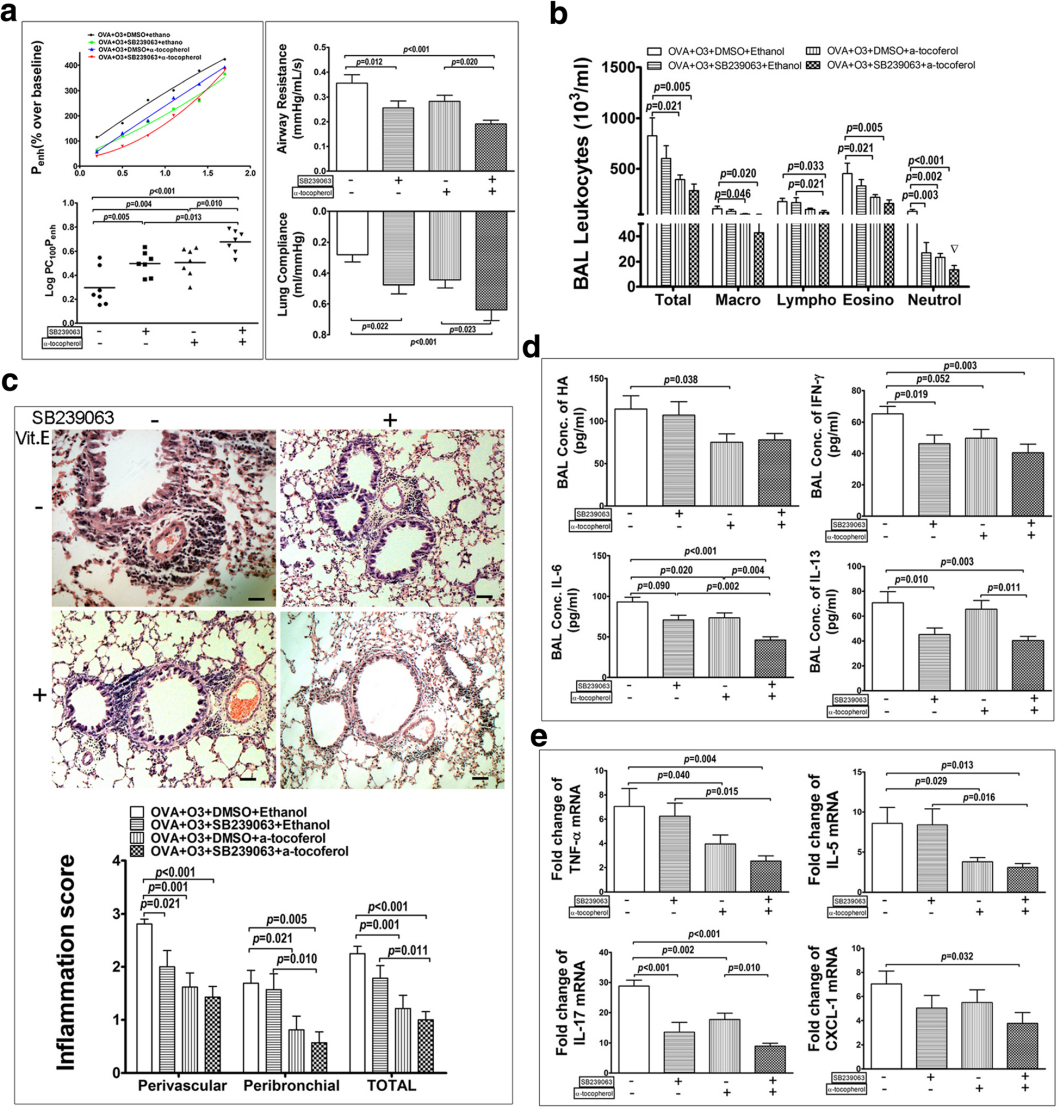
Role of p38 MAPK and oxidative stress in the ozonic effects on OVA challenged mice. OVA-O_3_ mice were received tail vein injection of SB239063 (4 mg/kg) (DMSO as control), or oral feeding of α-tocopherol (15 IU/kg) (50% ethanol as control), or both. **a** The lung function was assessed by the AHR in the left panels with the dose-response curves of P_enh_ (top) and the log concentrations of methacholine required to increase P_enh_ by 100% from baseline (LogPC_100_P_enh_) (bottom) showed, the airway resistance (top right) and the lung compliance (bottom right). **b** The total and differential cell counts in the BALF. **c** The representative images of H&E stained lung sections (top) and histological inflammation scores (bottom). **d** cytokines (IL-6, IL-13 and IFN-γ) and HA concentration in the BALF. **e** mRNAs expression of TNF-α, IL-5, IL-17 and CXCL-1 in the lungs. Data are shown as mean ± SEM; *n* = 7 in each group. Synergistic effect of SB239063& α-tocopherol, ▽:*p* < 0.05

Administration of SB239063 alone significantly decreased AHR (increased Log PC_100_Penh) and Raw, increased CL of mice in OVA-O_3_ model (Fig. 5a). Though administration of α-tocopherol alone on OVA-O_3_ group could significantly reduce the AHR, the inhibitory effects on the Raw and CL were not statistically significant (Fig. 5a). The combined administration of SB239063 and α-tocopherol decreased the AHR and Raw, and increased CL more than those treated with these inhibitors individually; however, no synergistic effect was observed (Fig. 5a).

Figure 5b showed the effect of SB239063 and/or α-tocopherol on OVA-O_3_ induced accumulation of inflammatory cells into the airway lumen. The total and differential cell counts revealed that SB239063 alone could significantly decreased the number of neutrophils in the BAL samples, but had little effect on the total cell counts and other types of cells (macrophages, lymphocytes and eosinophils). On the other hand, α-tocopherol alone could significantly decrease the number of total leukocytes and each sub-cell types except lymphocytes in the BAL samples. The co-administration of SB239063 and α-tocopherol had significant inhibitory effect on all cell counts, and particularly a synergistic inhibitory effect on the neutrophil accumulation.

Next, the histopathological role of p38 MAPK activation and oxidative stress generation on the ozonic effect was examined (Fig. 5c). As shown in both the histological images and the quantified inflammation score, administration of SB239063 alone significantly decreased the inflammation score in perivascular area, while α-tocopherol treatment alone significantly decreased the inflammation in both perivascular and peribronchial area, as well as in their average (total). The co-administration of SB239063 and α-tocopherol had profound effect on reducing the inflammation score in perivascular area, peribronchial area, and in total.

The effects of inhibition on p38 and oxidative stress in OVA-O_3_ model was further characterized by the cytokine profiles in BAL samples (Fig. 5d) and in the lungs (Fig. 5e). It was found that SB239063 treatment alone significantly decreased the OVA-O_3_ induced production of IFN-γ, IL-6 and IL-13 in the BAL fluid. Administration of α-tocopherol alone also reduced IFN-γ and IL-6, but not IL-13. The co-administration of SB239063 and α-tocopherol resulted in significant reduction in the level of all three cytokines with a profound effect on IL-6 production.

At the gene level, these two inhibitors alone or in combination also exhibited differential effects on down-regulating the expression of a different set of cytokines (TNF-a, IL-5, IL-17 and CXCL-1) in the lungs (Fig. 5e). Specifically, administration of SB239063 alone only significantly decreased IL-17 mRNA expression in the lungs. On the other hand, α-tocopherol treatment could significantly decrease the mRNA expressions of multiple cytokines, including TNF-α, IL-5 and IL-17, but not CXCL-1. Interestingly, only the co-administration of SB239063 and α-tocopherol was able to lower the CXCL-1 mRNA level with profound effect on all other cytokines (TNF-α, IL-5 and IL-17). Collectively, these data showed the potency of inhibiting both pathways in mitigating inflammation generated by O_3_ exposure in OVA challenged mice.

It is worth mentioning that hyaluronan, a type of glycosaminoglycan involved in inflammation, was differently modulated by the two chemical inhibitors (Fig. 5d). In comparison with the solvent treated OVA-O_3_ model, SB239063 treatment alone had no effect on hyaluronan level in the BALF, while α-tocopherol treatment alone significantly decreased the concentration of hyaluronan in the BAL fluid. Surprisingly, the decrease in hyaluronan level in the BAL fluid by the co-treatment of SB239063 and α-tocopherol was not statistically significant, indicating that the OVA-O3 induced hyaluronan production in the lungs was not p38 MAPK dependent.

## Discussion

In this study, we investigated how O_3_ exposure during the OVA challenge affects the asthma exacerbation in an OVA-allergic mouse asthma model. We found that O_3_ exposure during the OVA challenge process increased asthmatic inflammation in the airway and lungs, particularly promoting AHR and the airway resistance synergistically. We further demonstrated that p38 MAPK and oxidative stress play important roles in the observed ozonic effects on the asthma exacerbation.

Although there have been several similar mouse studies looking at the O_3_ effects on the asthmatic inflammation [10, 18, 22–24], our work is unique in the animal protocol to mimic the real situation of O_3_-induced asthma exacerbation in human. It is worth to note that the concentration of O_3_ used in this study (1.0 ppm) is relative higher than the atmosphere O_3_ concentration (~0.01 ppm) for inducing a measurable biological response. The major difference between our model and others was that the O_3_ exposure was applied during a different stage of immune establishment. Some groups conducted the exposure right after the OVA sensitization instead of during the challenge process [22], some introduced O_3_ exposure after the OVA challenge process was completed [18, 23, 24]. Though these studies did contribute to our understanding of the different perspectives of O_3_ effects in allergic asthma model, they are less relevant to the real-life situation of asthmatic patients undergoing O_3_ triggered exacerbation. It has been well-documented that the susceptibility to antigen challenge of asthmatic patients can be enhanced by the exposure to the ambient O_3_ [25–27]. Thus, applying the O_3_ exposure in the antigen challenge process, would better mimic the patient conditions of the ambient O_3_ induced asthma exacerbation. To date, there is only one study using a similar animal protocol of ours (i.e. O_3_ exposure during the process of antigen challenge), demonstrating that O_3_ promoted the eosinophilic airway and lung inflammation in the OVA-allergic mice [10]. Our study took one step forward to further address the specific ozonic effects on the AHR and lung mechanics (airway resistance Raw and lung compliance CL); more importantly, we defined the possible underlying mechanisms of these specific effects, suggesting that p38 MAPK and oxidative stress were critically involved in the process.

It has been reported that p38 MAPK pathway is involved in the O_3_-induced AHR and pulmonary inflammation in normal mice [11]. O_3_ exposure can activate p38 MAPK in the airway smooth muscle (ASM) of normal mice, and the phosphorylation of p38 MAPK will result in the phosphorylation of HSP27 (known as the p38 MAPK-HSP27 cascade), which eventually increases the contractility of ASM by enhancing its sensitivity to agonists, such as acetylcholine [12] and carbachol [28]. Our study further demonstrated that p38 MAPK-HSP27 pathway was also involved in the O_3_ induced asthma exacerbation as the phosphorylation of p38 MAPK and HSP27 was elevated in tracheal tissues in the OVA-O_3_ mouse model (Fig. 3). In addition, we also observed the profound increase in the AHR and Raw in the O_3_-exposed OVA-allergic asthma model (Fig. 2a). Note that the O_3_ exposure during OVA challenge could even cause a synergistic effect on p38 phosphorylation. Furthermore, inhibition of p38 MAPK with specific chemical inhibitor leads to the decrease in the AHR and Raw (Fig. 5a). Taken together, these data suggest that p38 MAPK-HSP27 cascade play an important role in the O_3_-induced elevation of the AHR and Raw in the allergic asthma model.

In addition to the p38 MAPK-HSP27 cascade, we also found that the oxidative stress is another key factor mediating the O_3_-enhanced AHR and Raw in the current OVA-O_3_ mouse model. First, the oxidative stress level in the lung tissues of O3-exposed normal mice was elevated as reflected by the MDA content and the activity of GSH-Px. Second, such increase, particularly in the GSH-Px activity, was further boosted by the synergistic effect from the O_3_ exposure and OVA challenge together. Third, the mitigation of oxidative stress by a ROS scavenger, α-tocopherol, led to the reduction of O_3_-enhanced AHR and Raw. All these evidences indicated that oxidative stress indeed contributed to the profound increase in AHR and Raw in mice of OVA-O_3_ model.

It has been shown that hyaluronans (low molecular weight, LMW) in the BALF of mice play essential roles in O_3_-induced enhancements in both AHR and the mucus production [18, 29]. In current study, we found that the hyaluronan in the BALF was synergistically increased by OVA challenge and O_3_ exposure; this effect could be inhibited by α-tocopherol alone but not the p38 inhibitor SB239063 (Fig. 5d), suggesting that oxidative stress is associated with the production of hyaluronan in the airway lumen. Consistently with this observation, previous study has demonstrated that the production of LMW-hyaluronan from the depolymerization of HMW-hyaluronan can be promoted significantly by ROS [30]. Furthermore, it has been reported that the mRNA expression of hyaluronan synthases (HAS1 and HAS2) was upregulated in a murine model of asthma [31], and the production of ROS can be detected within 2 h after the stimulation of ozone on airway epithelium [32]. Therefore, ozone exposure on asthma model could produce more LMW-hyaluronan than normal subjects, which offers an explanation for the synergistic effects of ozone and OVA on the change of BAL hyaluronan. Interestingly, in our previous study where the O_3_ was applied after the OVA challenge process was completed, we did not observe the synergistic effect of OVA and O_3_ on the hyaluronan production in BALF [18]. This is most likely because different protocols of O_3_ exposure were used. This also provides evidence for the importance of O_3_ exposure during the OVA challenge process.

Notably, we observed that the inhibition on oxidative stress in the OVA-O_3_ model could decrease the phosphorylation of p38 MAPK, but the inhibition on the p38 MAPK had no effect on the oxidative stress. Such inhibition by α-tocopherol on the p38 phosphorylation was much less than those caused by SB239063. In addition, α-tocopherol was incapable of inhibiting the downstream HSP27 phosphorylation as well as decreasing the Raw in the OVA-O_3_ mice. Together, these observations suggest that the oxidative stress pathway may probably be one of many upstream mediators of p38 MAPK activation in the OVA-O_3_ model. Though the underlying molecular mechanisms for oxidative stress induced p38 MAPK activation remain elusive, it is suggested by Williams, A. S. et al. that the Toll-like receptor (mainly TLR4 and TLR2) signaling pathways may be involved, as the p38 MAPK-mediated ozone-induced airway hyperresponsiveness was blocked by genetic inhibitions of TLR4 and TLR2 in mice [33]. Nevertheless, more detailed studies need to be conducted in the future to better understand this phenomenon.

In addition, our studies indicated that oxidative stress, but not p38 MAPK, contributed to the aggravation of allergic inflammation and immune responses by O_3_ exposure for the following reasons. First, we found that O_3_ exposure barely affected the inflammation in peribronchial area (Fig. 2c), thus, the peribronchial inflammation is mainly allergic in OVA-O_3_ model. We have shown that p38 MAPK inhibition did not reduce the peribronchial lung inflammation, however, the oxidative stress inhibition did (Fig. 5c), suggesting that the oxidative stress was involved in the production of O_3_-enhanced allergic peribronchial inflammation. Actually, this phenomenon has been previously described by Cook-Mills et al.*,* finding that α-tocopherol decreased the allergic lung inflammation in mice induced by the house dust mite [34]. Secondly, inhibition of oxidative stress was also found to inhibit the accumulation of eosinophils and the local production of IL-13 and IL-5 in the airway lumen of mice in OVA-O_3_ model. Similar findings were reported using rat model [35]. Mabalirajan, U. et al. found that the antioxidant treatment improved AHR and Th2 inflammatory response in an OVA-established asthma mouse model [17]. These findings suggested that the oxidative stress, but not the p38 MAPK, mediates the O_3_-enhanced allergic immune response.

Different from the eosinophils-mediated pulmonary allergic inflammation that is exclusively influenced by the oxidative stress in the current OVA-O_3_ model, the neutrophilic airway inflammation is attributed to both p38 MAPK activation and oxidative stress. Simultaneous inhibition of these two pathways exhibited synergistic effects on reducing the neutrophils infiltration to the lung (Fig. 5b). Consistently, lung mRNA expression of CXCL-1 (a main chemokine for neutrophil migration) was exclusively inhibited by the combined inhibition of both pathways. These results indicate a collaborative role of p38 MAPK and oxidative stress pathways in the O_3_-induced accumulation of neutrophils in the airway lumen of the OVA-allergic asthma model.

## Conclusions

O_3_ exposure during the OVA challenge process in an OVA-allergic asthma model elicits profound effects on many pathophysiological aspects in asthma exacerbation, including AHR, airway resistance, lung compliance and lung inflammation. A synergistic effect of O_3_ and OVA was found in the phosphorylation of p38 MAPK in the tracheal tissues and oxidative stress generation in the lung tissues. Using specific chemical inhibitors individually or in combination on these two pathways, we demonstrated that both p38 MAPK and oxidative stress play important roles in mediating the O_3_-induced pulmonary inflammation in OVA-allergic mice. Along with these findings, this study also offered a novel strategy to reverse the O_3_-elicited detrimental effects on asthma exacerbation by simultaneous inhibition of both the p38 MAPK and oxidative stress pathways. This inhibitory strategy may be applied to other oxidative stress-related inflammatory airway diseases, such as chronic obstructive pulmonary diseases, etc.

## Abbreviations

AHR: Airway hyperresponsiveness
BAL: bronchoalveolar lavage
CXCL: Chemokine (C-X-C motif) ligand
DMSO: Dimethylsulfoxide
GSH-Px: Glutathione peroxidase
HA: hyaluronan
HSP: Heat shock protein
IL: Interleukin
LogPC_100_P_enh_: The log concentrations of methacholine required to increase P_enh_ by 100% from baseline
MAPK: mitogen-activated protein kinase
MDA: Malondialdehyde
O_3_: Ozone
OVA: ovalbumin
P_enh_: enhanced pause
Vit.: Vitamin
